# Students’ voices: assessment in undergraduate clinical medicine

**DOI:** 10.11604/pamj.2020.36.130.22168

**Published:** 2020-06-25

**Authors:** Hanneke Brits, Johan Bezuidenhout, Lynette Jean van der Merwe, Gina Joubert

**Affiliations:** 1 Department of Family Medicine, School of Clinical Medicine, University of the Free State, Bloemfontein, South Africa,; 2 Health Sciences Education, Faculty of Health Sciences, University of the Free State, Bloemfontein, South Africa,; 3 Undergraduate Programme Management, School of Clinical Medicine, University of the Free State, Bloemfontein, South Africa,; 4 Department of Biostatistics, Faculty of Health Sciences, University of the Free State, Bloemfontein, South Africa

**Keywords:** Quality assessment, student opinions, clinical medicine, undergraduate medicine

## Abstract

**Introduction:**

the perception exists among students that not all clinical assessments in undergraduate medical programmes are of high quality. ‘Student voice’ is a term used to describe how students feel about and experience their education in a safe and controlled environment. This study aimed to investigate the opinions and experiences of medical students at the University of the Free State on the quality of assessment in the clinical phase of medicine.

**Methods:**

a cross-sectional study design was used. Quantitative data were collected with space to clarify opinions and make recommendations. The study population consisted of the clinical medical students in 2019 who had completed at least one module and one end-of-year assessment. Self-administered, anonymous questionnaires were distributed to obtain opinions and experiences regarding assessment. Questions in the questionnaire derived from an assessment framework for clinical medicine to ensure construct and content validity.

**Results:**

one hundred and ninety-two (192) students completed questionnaires (84.6% response rate). Less than half of the students were of the opinion that the assessments were fair, with lack of blueprinting and incorrect level of assessment major contributors to this opinion. Two thirds believed that the assessment was aligned with outcomes, however training was not aligned with the assessment. More than 90% of students reported on the lack of feedback after assessment. Valuable suggestions from the students included ways of assessing professionalism, timing of assessments and training of assessors.

**Conclusion:**

majority of students were of the opinion that there is room for improvement in the quality of assessment.

## Introduction

The cornerstones of good quality assessment are validity, reliability, fairness, feasibility, educational effect, acceptability, assessment of higher cognitive skills and benchmarking [1, 2]. It is important to be able to defend the quality of assessment in certifying courses e.g. medicine. In addition, students can give valuable information about their assessment experiences that may contribute to better assessment [3]. ‘Student voice’ is a term that describes the way students express how they feel and experience different aspects of education in a safe and controlled environment [4]. Youens and Hall [5] state that, ‘instead of treating students as voices crying in the wilderness, we would be far better served if we asked the voices’ owners what they think and listened actively to the answers′. Student opinions can be gathered through open forums, feedback slips, surveys and formal questionnaires [6]. Students’ opinions should be seen as viewpoints while landmark events, relationships and students’ gut reactions must be taken into consideration when these opinions are interpreted [7]. One experience (landmark event) may influence the way that a student responds to questions. For example if a student did not have enough time to complete the task at one of the 10 objective structured clinical examination (OSCE) stations the student passed through, the student may respond by stating that the time permitted for each OSCE station is insufficient [7]. Relationships, whether power relationships or personal relationships, between students and assessors may have a positive or negative effect on the way students express their opinions. Gee (2017) reports a student commenting: ‘I try to put what I feel is true and needs to be said, rather than worrying about what the people will think when they read it’ [7]. However, sometimes participants do not give any thought to their answers to questions and just complete the questionnaire as quickly as possible [7].

Several University of the Free State (UFS) undergraduate medical students who participated in the 2017 and 2018 end-of-year assessment expressed dissatisfaction with the marks they had obtained in clinical medicine (Personal communication with students). The phenomenon of student dissatisfaction with marks is not unique to the UFS, and is well described in the literature [8]. The motivation for this study was the perception that not all assessments in the clinical phase of the undergraduate medical programme at the UFS are of high quality. The clinical phase (Phase III) takes place over the last two and a half years of study, and entails rotations through various clinical departments in multiple health facilities. Clinical training takes place according to a fixed programme and for fixed periods. Formative assessment takes place throughout the clinical rotation and includes an end-of-block assessment. Together, these assessments contribute to a block mark (module mark), which gives students access to the final, summative, end-of-year assessment. Assessment in the clinical phase consists of theoretical assessment (multiple-choice questions and written questions) as well as practical assessments (long and short clinical cases and objective structured clinical assessments). This study aimed to investigate the opinions and experiences of students regarding the quality of the assessment they experienced in the clinical phase of the undergraduate medical programme.

## Methods

A cross-sectional study design was used. Quantitative data were collected with space to clarify opinions. The study population consisted of all the students who had completed at least one module and one end-of-year assessment in the clinical phase of the undergraduate medical programme. All 227 students in the fourth and fifth years of the undergraduate medical programme at the UFS during 2019 were included in the study sample. The steps of questionnaire development, as described by Katzenellenbogen and Joubert, [9] were used to design the questionnaire. Questions included in the questionnaire derived from a framework to benchmark the quality of assessment in undergraduate clinical medicine [10]. Questions were grouped under headings, to structure the responses. These headings were principles of quality assessment (fairness, validity, reliability and educational effect), assessment methods, assessment of soft skills and recommendations on how to improve assessments. Responses were mainly yes or no followed by a question to justify or expand on the responses. A question on how to improve assessment was also included. The input of the co-authors was used to improve the validity and reliability of the questionnaire. For internal validity, the face validity of the questionnaires was tested in a pilot study conducted on five interns who had completed their studies at the UFS in 2018. No items were changed, but the layout was adjusted to improve flow. The questions in the questionnaires derived from the literature, to ensure construct and content validity. The researcher tried to be as objective as possible during data interpretation and representation. Numbers were double checked and responses were quoted verbatim under each heading.

McMillan and Schumacher [11] describe questionnaires as research instruments that can be used to gather information on the current statuses of a situation from a specific population. A self-administered, anonymous questionnaire was used for data collection, as it posed less of a threat of exposure to participants [12]. After the purpose of the questionnaire had been explained to the participants, student group leaders distributed the printed questionnaires and information leaflets to the students. After completion the questionnaires were returned to the group leaders and then to the researcher. Data was transferred to Excel data sheets by the first author twice, to check for integrity. Percentages were calculated for different responses per year group. Responses and the justification for answers were grouped by the first author according to question headings.

**Ethical considerations:** ethical approval for the study was obtained from the Health Sciences Research Ethics Committee, UFS (UFS-HSD 2019/0001/2304). UFS authorities approved the inclusion of medical students. Participation was voluntary, with implied consent being given by completing the anonymous questionnaires. Participants did not receive any compensation for completing the questionnaires and there was no penalty for not participating in the study.

## Results

A total of 108 out of 119 fourth-year students (90.6%), and 84 out of 106 fifth-year students (79.2%) returned completed questionnaires. More than 80% of students gave justifications for responses and 75.5% made recommendations to improve assessment practices.

**Quality of assessment:** only 43.5% of fourth-year and 44.1% of fifth-year medical students felt that the current assessment is fair. In Table 1 the percentages of students who agreed with the statements are displayed. Open responses were not split for different year groups. About half the students clarified their responses in relation to the alignment of questions with outcomes in tests and exams; the following are some of the comments students made: ‘The outcome is in the book, but they don’t train that and then they ask that.’ ‘Because we spend too little time with patients, we don’t see all the things that they ask in clinical cases, although it is mentioned in the module guides.’ ‘We’re not assessed on what we see commonly.’ ‘We know the outcomes from our module guides.’ Many students had opinions regarding the spread of questions and the following comments were made: ‘You know who compiled the questions, because they only concentrate on their own field of expertise.’ ‘We need more assessments, to cover more work.’ ‘Some subjects ask 10 to 15 marks from 20 chapters, it’s not fair.’ ‘With MCQs (*multiple choice questions*) you get a good spread of questions, but they are not necessarily important.’ ‘It is dependent on the subject, some do it better than others. OSCEs help.’ More than 80% of students commented that the level of assessment was on specialist level, rather than on general practitioner level. The following were comments regarding the difficulty of questions: ‘We’re not specialists, don’t assess us as specialists.’ ‘No need for specialist special investigations to be tested.’ ‘They don’t assess hard work and professionalism.’ ‘They expose us to specialist work and then they ask that, we’re not specialists.’ ‘Sometimes you’re lucky and get common things.’

**Table 1:** percentages of fourth and fifth year students who answered yes to some of the questions on the quality of assessments

STATEMENT	4th year	5th year
Do you think that the questions in tests/exams are aligned with the outcomes of the programme?	63.0%	67.9%
Do you think that the questions are spread to cover all work? (Blueprinting)	35.2%	45.2%
Do you think that there is a good spread between easy and difficult questions? (Bloom´s taxonomy)	52.8%	67.9%
Do you think that appropriate assessment methods are used in tests/exams?	66.7%	67.9%

**Assessment methods:** regarding the appropriateness of assessment methods, most students commented negatively about multiple-choice questions as an assessment method. ‘They (*multiple-choice questions*) don’t test knowledge.’ ‘Questions not up to standard, they use the same old questions and things changed.’ ‘MCQs can’t test arguments.’ ‘You get your marks quickly, but don’t know the correct answers.’ A third of students made recommendations about assessment methods. The following recommendations were made: ‘Improvement is necessary, but I don’t know how.’ ‘Please include short questions, so that I can explain.’ ‘We should get exposure to the exam assessments during rotations and block assessments.’ ‘Written OSCES are not clear and not clinical, don’t use them.’ Ten responses were received regarding assessors and they were all negative. Some of these were: ‘Train the examiners.’ ‘Your marks are dependent on the examiner, they like you or they don’t. It’s not fair if they don’t like you.’ ‘Some people give very poor marks, they think that 65% is good.’ According to the students, feedback is almost non-existent. Table 2 indicates percentages of students of the two years who agreed with certain statements. More than 75% of students commented on feedback, or the lack of feedback. Most students asked for constructive feedback to assist with learning. Some comments were the following: ‘Formal feedback sessions should be scheduled like in Phases 1 and 2’. ‘Immediate feedback after clinical cases will help a lot. Then you know how to improve.’ ‘By the time that you get your marks, you can’t even remember what they asked and what you answered.’ ‘Please fill in an assessment form for clinical assessments and give a copy to the student after assessment. Then the student will see how to improve (*and*) the lecturer how to give marks.

**Table 2:** percentage of fourth and fifth year students who answered yes to questions on feedback

STATEMENT	4th year	5th year
Assessment results are available within 2 weeks of the assessment	35.2%	24.1%
Memorandums are available for assessments	2.8%	7.1%
We receive formal feedback after assessments	7.4%	9.5%

**Assessment of soft skills:** almost all students, 85.2% of fourth years and 92.9% of fifth years, agreed that soft skills, such as professionalism, should be assessed. Practical suggestions for assessment of these skills included using cellular phone applications to assess group members weekly, asking patients and other health care professionals for feedback, and that the responsible registrar provides feedback after the rotation. Twenty percent of students commented on the positive or negative effects that role models could have on professional behaviour. Most value good role models, but they could also learn from bad role models, as indicated by this response: ‘At least we can see how not to behave, if we look at some people.’

**Competence and assessment:** most students thought that assessment results were not an indication of competence. Table 3 displays these results. Only 48.1% of fourth years, compared to 78.6% of fifth-year students, felt confident about their skills even if they had passed an assessment. Many students mentioned the lack of feedback after assessment as a reason for their lack of confidence. Another reason mentioned was that the marks that they had obtained were not necessarily an indication of their competence. Some comments in this regard were: ‘The doctor said that I sucked and then gave me 65%. For me that is good.’ ‘They said that I did well, but I only just passed.’ Comments in relation to this topic included: ‘You should know from your block assessments if you will pass, but it’s not so.’ ‘Some students are good with exams, but not competent.’ ‘If I pass I’m good, not sure about others.’ ‘If I pass they (*the assessments*) are good, if I fail they are not.’

**Table 3:** percentage of fourth and fifth year students who answered yes to questions on competence and assessment

STATEMENT	4th year	5th year
My end-of-block assessment mark is a good predictor of my end-of-year assessment mark.	26.9%	42.9%
Students who pass the final assessment in 5th year are competent to become interns.	38.9%	41.7%
Students who fail the final assessment in 5th year are not competent to become interns and need more training.	15.7%	8.3%

**Recommendations to improve clinical assessment:** students made recommendations to improve the type of assessments, examiners, the examination process and the content of the assessment, as well as general recommendations.

**The type of assessment:** students recommended continuous clinical assessment, next to patients or in clinical areas, with immediate feedback. More than 70% of students indicated that multiple-choice questions alone are not enough to test knowledge, and they wanted short questions, or short questions in addition to multiple-choice questions. Students felt strongly that end-of-block assessments should be sufficient to test competence, and that they do not need to do a final assessment at the end of the year, on the same subjects again. Students also recommended that assessment during the clinical blocks should promote them to an integrated assessment at the end of the final year. ‘Give us one MIMA (*integrated medical assessment*) OSCE at the end of the year, like in second and third year.’

**Examiners:** only a few students made recommendations regarding examiners, among which, ‘Train the examiners, some are clueless.’ ‘Some examiners should not examine, first examine them.’ ‘Use external examiners from other departments.’ ‘We need better role models as examiners.’

**The examination process:** many students recommended that continuous assessment takes place while patients are presented in clinical areas, and written feedback given. Another recommendation was to record presentations, which may assist students and examiners to clarify areas of disagreement or improvement. Students requested exposure to mock assessments, to prepare them for and to assist them in their preparation for final assessments. The timing of assessments is important: it should not be after long calls, or late in the day, when students and examiners are tired.

**Content of assessments:** about 30% of students believed that their training was not aligned with the assessment. ‘We must spend more time in the clinics and wards, then we will learn more and do better in exams.’ In contrast, students also commented as follows: ‘They ask what is easy to mark, not what they teach us.’ ‘They use the same patient, and it’s not fair if you are the first or last student.’ ‘Some patients are good to use in exams, but it’s not really what we need to know.’ Most students indicated that assessment should cover general conditions, and not specialist or super-specialist conditions. Some students also provided general recommendations about how better training may affect assessment. ‘Everybody must train the same facts from prescribed references.’ ‘Different disciplines do things differently, they should have a uniform format, for instance when presenting patients.’ ‘Tutorials and discussions are much better than lectures. We can read better than some lecturers.’ ‘Use the good role models to train other lecturers.’

## Discussion

The response rate of a survey matters, and a good response rate indicates that the researcher can generalize the results for the population under investigation [3]. The response rate of 85% in this survey makes the results obtained generalizable, as they represent the opinions of current clinical medical students at the UFS. It may also indicate that students were eager to voice their opinions. Less than half the students believed that current assessments were fair. They thought that the questions were too difficult for undergraduate students, and they were not satisfied with the spread of questions (blueprinting). This opinion of the students may be because specialists and super specialists conduct training in the undergraduate and postgraduate medical programme, and may find it difficult to accept that undergraduate students need only limited knowledge of ‘their’ subject. McConlogue [13], Price *et al*. [14] and Yorke [15] describe how complex it is to set exams when different dimensions must be considered. In summative assessment, blueprinting is problematic, because of the large volumes of work that must be covered by a single assessment; this is even more complex when clinical assessment involves patients and different assessment sites. Sites may differ regarding resources and disease profiles, and the conditions of patients may also change during or between assessments. Although >90% of students reported not receiving feedback after assessments, they value feedback and gave good suggestions to improve the lack of feedback. The value of formative feedback is that it assists students to measure themselves and ‘identify the gap between their current and desired performance’ [16]. To get the maximum advantage of feedback, it should be of good quality, specific and on time [17].

The Health Professions Council of South Africa describes core competencies for undergraduate medical students, including professionalism [18]. Most students wanted a formal assessment of ‘soft skills’, and suggested peer and patient assessment. It is concerning that they do not consider all lecturers and doctors to be good role models of professional behaviour. Less than half of fourth years, and more than three quarters of fifth-year students were confident about their competence after passing an assessment. In the fifth year group, this confidence may be the result of more experience with clinical assessment, and being better prepared for assessment. Most students expressed that assessment without feedback does not provide a good indication of competence or incompetence. An interesting finding is that students were satisfied with the assessment if they passed, but not when they had failed. The students also expressed different views on assessments in which they were involved and assessments involving others. A study in the United States found that medical students were able assess their own knowledge, skills and behaviour on assessment accurately, with fewer than 10% of students overrating their knowledge [19]. However, students could not accurately evaluate others, and, therefore, student assessments lack validity and reliability [20-22]. Students provided a number of recommendations on how the current assessment could be improved. A limitation of this study is that it reports opinions and experiences, rather than facts which are noted. It is also possible that despite the pilot study some students did not understand the terms used in the questionnaire, nevertheless, they answered all the questions.

## Conclusion

The students provided valuable feedback on their experiences of the current assessment in clinical medicine. Fifth-year students were more satisfied with their assessment than fourth years. Students had different and sometimes contrasting opinions on assessment of themselves and others. These results and the recommendations made by students will be discussed at appropriate forums, with the aim of improving the quality of assessment. Students will also receive feedback on this research to encourage transparency of the process. Majority of students were of the opinion that there is room for improvement in the quality of assessment.

### What is known about this topic

- The quality of clinical assessment should be established and defendable;
- Student opinions are valuable and reliable sources of information.

### What this study adds

- Misalignment between outcomes, training and assessment;
- Recommendations on assessment methodology e.g. integrated assessments;
- Students value feedback.
